# A new cadmium coordination polymer based on 4-amino-4*H*-1,2,4-triazole

**DOI:** 10.1107/S2056989018000464

**Published:** 2018-01-12

**Authors:** Maha Said, Habib Boughzala

**Affiliations:** a Laboratoire de Matériaux et Cristallochimie, Faculté des Sciences de Tunis, Université de Tunis El Manar, 2092 Manar II Tunis, Tunisia

**Keywords:** crystal structure, hybrid coordination polymer, cadmium(II), triazole

## Abstract

A new cadmium coordination polymer, poly[bis­(4-amino-4*H*-1,2,4-triazolium) [bis­(μ_2_-4-amino-4*H*-1,2,4-triazole-κ^2^ *N* ^1^:*N* ^2^)tetra-μ_2_-chlorido-tetra­chlorido­tri­cad­mium(II)] dihydrate], {(C_2_H_5_N_4_)_2_[Cd_3_Cl_8_(C_2_H_4_N_4_)_2_]·2H_2_O}_*n*_, was synthesized by the reaction of 4-amino-4*H*-1,2,4 triazole with cadmium(II) chloride in aqueous solution. With an unusual architecture, the crystal structure exhibits two distorted octa­hedral coordinations of Cd^II^ joined by edge sharing. The first is composed by four chlorine and two N atoms from the triazole ligands. The second is formed by five Cl atoms and by one N atom from the triazole ligand. The charge of the resulting two-dimensional anionic framework is balanced by the organic triazole cations. The lattice water mol­ecules form a network of hydrogen bonding. N—H⋯Cl and π–π stacking inter­actions are also involved in the supra­molecular network stability.

## Abstract 2

Here we report the chemical synthesis and crystal structure of a new hybrid chloro-cadmium coordination polymer based on 4-amino-4*H*-1,2,4 triazole solved by single-crystal X-ray diffraction. With an unusual architecture, the crystal structure exhibits two distorted octa­hedral coordinations of Cd^II^ joined by edge sharing. The first is composed by four chlorine and two N atoms from the triazole ligands. The second is formed by five Cl atoms and by one N atom from the triazole ligand.

## Chemical context   

The last decade has seen a large number of investigations of Cd^II^ hybrid coordination polymers (HCPs). Indeed, these materials exhibit a wide variety of polymeric frameworks with attractive properties. The coordination sphere of Cd^II^ is variable, with coordination numbers ranging from four to eight, corresponding to different geometries (tetra­hedral, square planar, square pyramidal, trigonal bipyramidal, octa­hedral, penta­gonal bipyramidal, bicapped triangular prismatic and dodeca­hedral; Li & Du, 2011 ▸). Many factors should be considered in the self-assembly processes of HCPs, such as the nature of the organic ligands, temperature, pH values, solvents, and so on (Guo *et al.*, 2013 ▸). The choice of the organic ligands is an important factor that greatly influences the structure and stabilization of the coordination architecture formed (Tao *et al.*, 2000 ▸; Choi & Jeon, 2003 ▸). In this regard, organic building units that are based on five-membered N-heterocycles such as 1,2,4 triazole exhibit a strong and typical property of acting as bridging ligands between two metal centres. These bridges can adopt various different geometries, depending on the donor atoms of the ligand and the properties of the metal (Haasnoot *et al.*, 2000 ▸). The reaction of 4-amino-4*H*-1,2,4 triazole (NH_2_trz) with cadmium dichloride leads to the formation of the title two-dimensional coordination polymer.

## Structural commentary   

**Figure 1:**
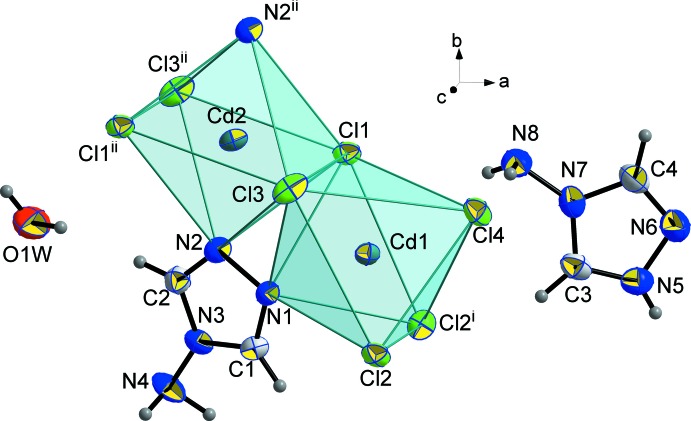
*ORTEP* of the asymmetric unit of the studied compound plus the atoms necessary to complete the coordination around the Cd ions. Cd2 is on the special position (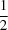, 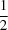, 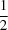). Displacement ellipsoids are drawn at the at the 50% probability level. [Symmetry codes: (i) *x*, 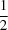 − *y*, −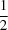 + *z*; (ii) 1 − *x*, 1 − *y*, 1 − *z*.]

The asymmetric unit of the studied compound, completed by the atoms necessary to achieve the coordination around the Cd ions, is represented in Fig. 1 ▸. It comprises one and a half Cd^II^ cations [with Cd2 occupying the special position (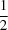, 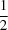, 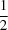)], one triazole mol­ecule (NH_2_trz), one triazolium cation (NH_2_trzH)^+^, four chloride anions and one lattice water mol­ecule. Cd1 and Cd2 are bridged by the coordinated triazole mol­ecule (NH_2_trz) through atoms N1 and N2, and by the two chlorine atoms Cl1 and Cl3.
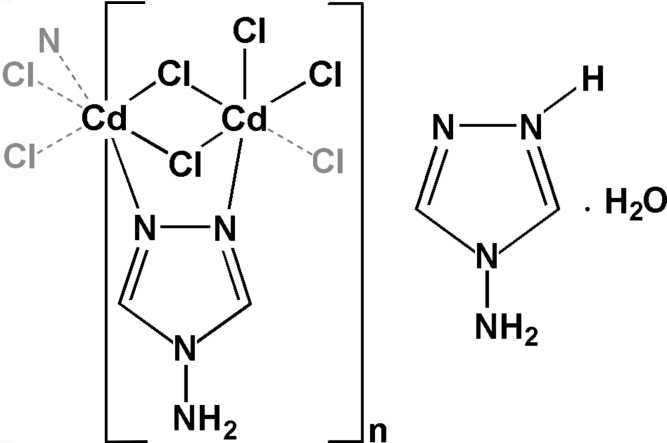


Both metals show an octa­hedral coordination geometry. Cd1 is surrounded by the five chloride anions Cl1, Cl2, Cl3, Cl4, Cl2^i^ [symmetry code: (i) *x*, 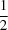 − *y*, *z* − 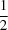] and the nitro­gen N1 of the coordinated triazole ring (NH_2_trz). On the other hand, Cd2 is bonded to four equatorial chloride anions (Cl1, Cl3, Cl1^ii^ and Cl3^ii^) and two axial nitro­gen atoms, N2 and N2^ii^, belonging to the coordinated triazole (NH_2_trz) and to its symmetry-related analogue, respectively [symmetry code: (ii) 1 − *x*, 1 − *y*, 1 − *z*). As a result of the bridge formed by atoms N1 and N2 of the triazole ligand, the Cd1⋯Cd2 distance is 3.6145 (7) Å. Selected geometrical parameters are summarized in Table 1 ▸, showing that the octa­hedron around Cd1 is more distorted than the one around Cd2.

**Figure 2:**
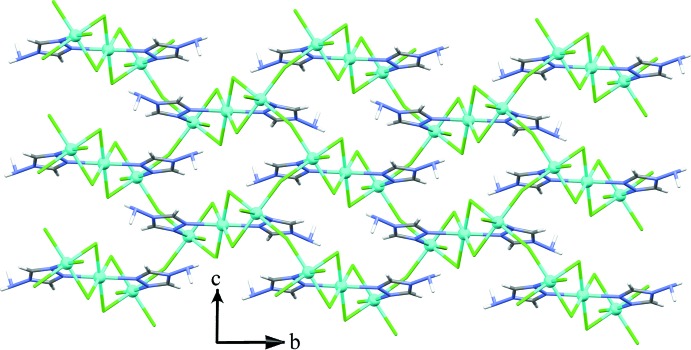
Crystal packing showing the two-dimensional anionic framework of the title compound.

**Figure 3:**
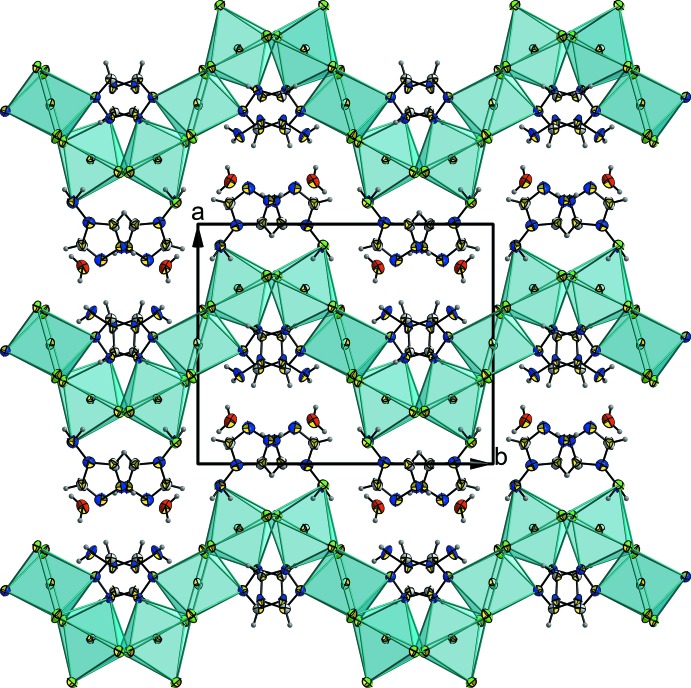
Corrugated anionic sheets with the non-coordinating triazolium cations and water mol­ecules located in the inter­layer space. Displacement ellipsoids are drawn at the 50% probability level.

When symmetry is applied, a Cd_3_Cl_8_(NH_2_trz)_2_ building block is formed. These trinuclear units are connected *via* the chloride ions Cl2 to build up infinite inorganic corrugated sheets in the *bc* plane, stacked along the *a*-axis direction (Fig. 2 ▸). The triazolium cations (NH_2_trzH)^+^ and the water mol­ecules are located in the inter­layer space (Fig. 3 ▸), inter­acting with the anionic framework by hydrogen bonds. Thus, the overall three-dimensional network consists of alternate organic–inorganic hybrid layers, responsible for the inter­esting behaviour of this class of materials.

## Supra­molecular features   

The crystal structure of the title compound is mainly stabilized by hydrogen-bonding and π–π stacking inter­actions. In particular, a number of O—H⋯Cl, O—H⋯N, N—H⋯O and N —H⋯Cl hydrogen bonds is present (Table 2 ▸), involving the lattice water mol­ecules, the triazolium cations, the organic ligands and the chlorine anions. These hydrogen bonds connect the organic and inorganic moieties, leading to a self-organized, hydrated hybrid structure.

**Figure 4:**
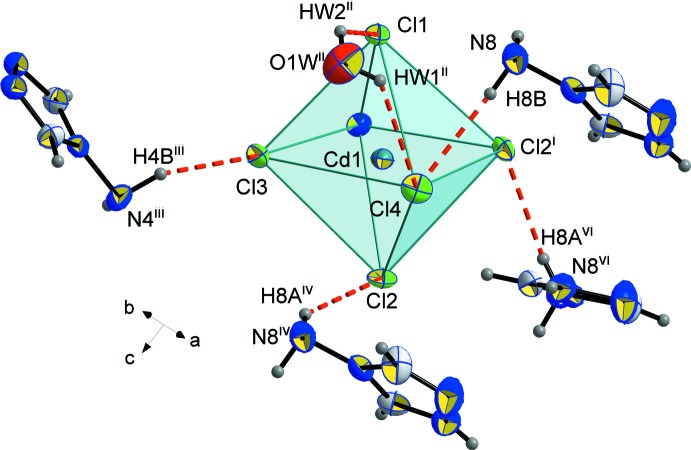
Hydrogen bonds (red dashed lines) involving the chloride anions around Cd1. Displacement ellipsoids are displayed at the 50% probability level. [Symmetry codes: (i) *x*, 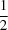 − *y*, −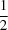 + *z*; (ii) 1 − *x*, 1 − *y*, 1 − *z*; (iii) 1 − *x*, 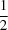 + *y*, 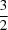 − *z*; (iv) *x*, *y*, 1 + *z*; (vi) *x*, 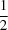 − *y*, 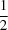 + *z*.]

The chloride anions around Cd1 and Cd2 form hydrogen bonds both with the amine H atoms of the (NH_2_trz) ligands and with the H atoms of the water mol­ecules (Figs. 4 ▸ and 5 ▸; Table 2 ▸): Cl1⋯H*W*2^ii^—O1*W* ^ii^, Cl3⋯H4*B* ^iii^—N4^iii^, Cl4⋯H*W*1^ii^—O1*W* ^ii^, Cl4⋯H8*B*-N8, and Cl2⋯H8*A* ^iv^—N8^iv^ [symmetry codes: (iii) 1 − *x*, 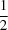 + *y*, 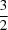 − *z*; (iv) *x*, *y*, 1 + *z*].

**Figure 6:**
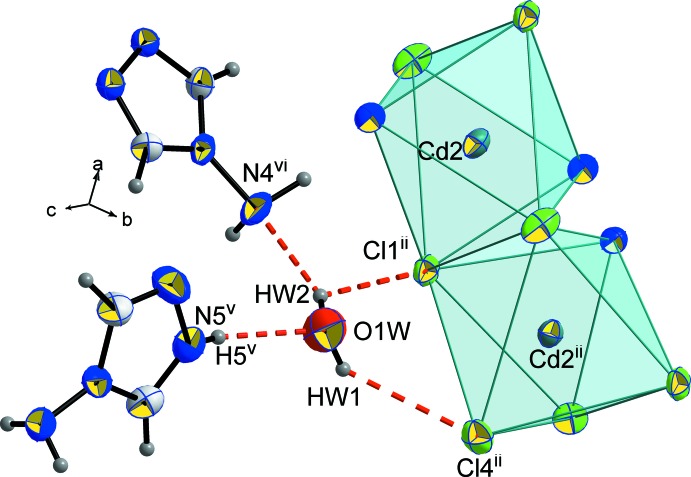
The hydrogen-bonding inter­actions around a single water mol­ecule involving the chlorine atoms, the (NH_2_trz) ligand and the (NH_2_trzH)^+^ cation. Displacement ellipsoids are displayed at the 50% probability level. [Symmetry codes: (ii) 1 − *x*, 1 − *y*, 1 − *z*; (v) −1 + *x*, 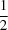 − *y*, 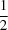 + *z*; (vi) *x*, 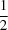 − *y*, 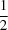 + *z*.]

Besides forming hydrogen bonds with the chloride anions Cl1 and Cl4, the water mol­ecules also inter­act with the triazole ligands and with the lattice triazolium cations, acting as acceptor and donor, respectively (Fig. 6 ▸ and Table 2 ▸): O1*W*⋯H5^v^–N5^v^ and N4^vi^⋯H*W*2—O1*W* [symmetry codes: (v) *x* − 1, 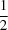 − *y*, 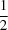 + *z*; (vi) *x*, 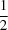 − *y*, *z* + 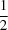].

Finally, the coordinated triazole rings (NH_2_trz) are connected along the *c*-axis direction through π–π stacking inter­actions, with a centroid–centroid distance of 3.761 (7) Å.

## Database survey   

Recently, a great deal of attention has been paid to the rational design and synthesis of new hybrid coordination polymers (HCPs) composed of metal ions and bridging ligands due to their fascinating structural diversity and their potential application as functional materials (Xiong *et al.*, 2001 ▸; Liao *et al.*, 2004 ▸; Gao *et al.*, 2008 ▸). These coordination polymers exhibit a wide range of infinite zero- to three-dimensional frameworks with inter­esting structural features, which result from coordination bonding, hydrogen-bonding and aromatic π–π stacking inter­actions as well as van der Waals forces (Su *et al.*, 2003 ▸).

A search of the latest version of the Cambridge Structural Database (Version 5.38; Groom *et al.*, 2016 ▸) based on the organic fragment ‘4-amino-4*H*-1,2,4-triazole’ of the studied compound yielded 70 hits. The structure of the chloro-cadmate PEPWIR (Zhai *et al.*, 2006 ▸) is probably the nearest to that of the title compound, even if it lacks the water mol­ecules of crystallization and the protonated triazole cations. This is probably due to the difference in the stoichiometry of the initial reagents and to the solvent used in the chemical synthesis. Two other related compounds comprising 4-amino-4*H*-1,2,4-triazole in combination with chloride ligands are the coordination polymer ROFJED (Wang *et al.*, 2014 ▸) and the discrete complex GAVFEP (Xuan-Wen, 2005 ▸).

## Synthesis and crystallization   

The compound was prepared by the reaction of 4-amino-4*H*-1,2,4 triazole and CdCl_2_·H_2_O (molar ratio 1:1) in an equal volume of water and ethanol (10 ml) mixed with 2 ml of hydro­chloric acid (37%). The solution was stirred for 1 h. Colourless crystals suitable for X-ray diffraction were grown in two weeks by slow evaporation at room temperature.

## Refinement   

Crystal data, data collection and structure refinement details are summarized in Table 3 ▸. Atoms H1, H2 and H3 were placed in calculated positions and refined using a riding model: C—H = 0.93 Å with *U*_iso_(H) = 1.2*U*_eq_(C). The other hydrogen atoms were found in the difference-Fourier map. The coordinates of H8*A*, H8*B* and H4*A* of the amine terminal groups were kept fixed, with *U_i_*_so_(H)= 0.05.

## Supplementary Material

Crystal structure: contains datablock(s) I. DOI: 10.1107/S2056989018000464/xi2006sup1.cif

Structure factors: contains datablock(s) I. DOI: 10.1107/S2056989018000464/xi2006Isup2.hkl

CCDC reference: 1810807

Additional supporting information:  crystallographic information; 3D view; checkCIF report

## Figures and Tables

**Figure 5:**
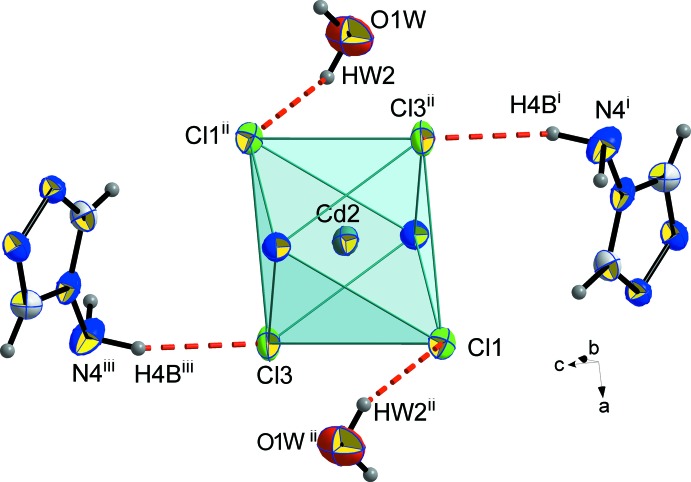
Hydrogen bonds (red dashed lines) involving the chloride anions around Cd2. Displacement ellipsoids are displayed at the 50% probability level. [Symmetry codes: (i) *x*, 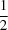 − *y*, −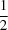 + *z*; (ii) 1 − *x*, 1 − *y*, 1 − *z*; (iii) 1 − *x*, 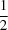 + *y*, 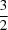 − *z*.]

**Table 1:** Selected geometric parameters (Å, °)

Cd1—N1	2.365 (4)	Cd1—Cl1	2.6769 (14)
Cd1—Cl4	2.5120 (14)	Cd2—N2	2.393 (5)
Cd1—Cl2	2.6148 (13)	Cd2—Cl3	2.5874 (16)
Cd1—Cl3	2.6418 (14)	Cd2—Cl1	2.6332 (14)
Cd1—Cl2^i^	2.6754 (13)		
			
N1—Cd1—Cl4	174.37 (11)	Cl3—Cd1—Cl1	84.86 (4)
Cl2—Cd1—Cl1	174.60 (4)	Cl3—Cd2—Cl1	86.85 (5)

Symmetry code: (i) 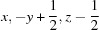.

**Table 2:** Hydrogen-bond geometry (Å, °)

*D*—H⋯*A*	*D*—H	H⋯*A*	*D*⋯*A*	*D*—H⋯*A*
O1*W* ^ii^—H*W*2^ii^⋯Cl1	0.86 (6)	2.68 (7)	3.239 (6)	124 (6)
N4^iii^—H4*B* ^iii^⋯Cl3	1.00 (8)	2.60 (7)	3.399 (5)	136 (5)
N8^iv^—H8*A* ^iv^⋯Cl2	0.85	2.64	3.370 (5)	144
O1*W* ^ii^—H*W*1^ii^⋯Cl4	0.86 (7)	2.67 (8)	3.319 (6)	134 (8)
N8—H8*B*⋯Cl4	0.90	2.53	3.423 (5)	172
N5^v^—H5^v^⋯O1*W*	0.75 (8)	1.97 (8)	2.649 (8)	151 (8)
O1*W*—H*W*2⋯N4^vi^	0.86 (6)	2.44 (6)	3.247 (9)	157 (6)

Symmetry codes: (ii) 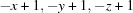; (iii) 
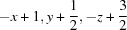
; (iv) 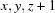; (v) 
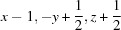
; (vi) 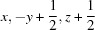.

**Table 3:** Experimental details

Crystal data	
Chemical formula	(C_2_H_5_N_4_)_2_[Cd_3_Cl_8_(C_2_H_4_N_4_)_2_]·2H_2_O
*M*_r_	995.21
Crystal system, space group	Monoclinic, *P*2_1_/*c*
Temperature (K)	298
*a*, *b*, *c* (Å)	12.685 (3), 15.498 (3), 7.375 (2)
β (°)	97.12 (3)
*V* (Å^3^)	1438.6 (6)
*Z*	2
Radiation type	Mo *K*α
μ (mm^−1^)	2.98
Crystal size (mm)	0.71 × 0.21 × 0.21

Data collection	
Diffractometer	Enraf–Nonius CAD-4
Absorption correction	ψ scan (North *et al.*, 1968 ▸)
*T*_min_, *T*_max_	0.799, 1.000
No. of measured, independent and observed [*I* > 2σ(*I*)] reflections	3670, 3136, 2654
*R*_int_	0.032
(sin θ/λ)_max_ (Å^−1^)	0.638

Refinement	
*R*[*F* ^2^ > 2σ(*F* ^2^)], *wR*(*F* ^2^), *S*	0.040, 0.123, 1.06
No. of reflections	3136
No. of parameters	190
No. of restraints	5
H-atom treatment	H atoms treated by a mixture of independent and constrained refinement
Δρ_max_, Δρ_min_ (e Å^−3^)	1.58, −1.99

Computer programs: *CAD-4 EXPRESS* (Enraf–Nonius, 1994 ▸), *XCAD4* (Harms & Wocadlo, 1995 ▸), *SHELXS97* (Sheldrick, 2008 ▸), *SHELXL2014* (Sheldrick, 2015 ▸), *DIAMOND* (Brandenburg, 2006 ▸) and *publCIF* (Westrip, 2010 ▸).

## supplementary crystallographic information

### Crystal data

**Table:**

(C_2_H_5_N_4_)_2_[Cd_3_Cl_8_(C_2_H_4_N_4_)_2_]·2H_2_O	*F*(000) = 956
*M**_r_* = 995.21	*D*_x_ = 2.298 Mg m^−^^3^
Monoclinic, *P*2_1_/*c*	Mo *K*α radiation, λ = 0.71073 Å
*a* = 12.685 (3) Å	Cell parameters from 25 reflections
*b* = 15.498 (3) Å	θ = 10–15°
*c* = 7.375 (2) Å	µ = 2.98 mm^−^^1^
β = 97.12 (3)°	*T* = 298 K
*V* = 1438.6 (6) Å^3^	Prism, colourless
*Z* = 2	0.71 × 0.21 × 0.21 mm

### Data collection

**Table:**

Enraf–Nonius CAD-4 diffractometer	*R*_int_ = 0.032
Radiation source: Enraf Nonius FR590	θ_max_ = 27.0°, θ_min_ = 2.1°
non–profiled ω/2τ scans	*h* = −16→16
Absorption correction: ψ scan (North *et al.*, 1968)	*k* = −19→1
*T*_min_ = 0.799, *T*_max_ = 1.000	*l* = −9→1
3670 measured reflections	2 standard reflections every 120 min
3136 independent reflections	intensity decay: 8%
2654 reflections with *I* > 2σ(*I*)	

### Refinement

**Table:**

Refinement on *F*^2^	Hydrogen site location: mixed
Least-squares matrix: full	H atoms treated by a mixture of independent and constrained refinement
*R*[*F*^2^ > 2σ(*F*^2^)] = 0.040	*w* = 1/[σ^2^(*F*_o_^2^) + (0.0848*P*)^2^ + 1.0345*P*] where *P* = (*F*_o_^2^ + 2*F*_c_^2^)/3
*wR*(*F*^2^) = 0.123	(Δ/σ)_max_ = 0.001
*S* = 1.06	Δρ_max_ = 1.58 e Å^−^^3^
3136 reflections	Δρ_min_ = −1.99 e Å^−^^3^
190 parameters	Extinction correction: SHELXL2014 (Sheldrick, 2015), Fc^*^=kFc[1+0.001xFc^2^λ^3^/sin(2θ)]^-1/4^
5 restraints	Extinction coefficient: 0.0041 (7)

### Special details

**Table:**

Geometry. All esds (except the esd in the dihedral angle between two l.s. planes) are estimated using the full covariance matrix. The cell esds are taken into account individually in the estimation of esds in distances, angles and torsion angles; correlations between esds in cell parameters are only used when they are defined by crystal symmetry. An approximate (isotropic) treatment of cell esds is used for estimating esds involving l.s. planes.
Refinement. Refinement of F^2^ against ALL reflections. The weighted R-factor wR and goodness of fit S are based on F^2^, conventional R-factors R are based on F, with F set to zero for negative F^2^. The threshold expression of F^2^ > 2sigma(F^2^) is used only for calculating R-factors(gt) etc. and is not relevant to the choice of reflections for refinement. R-factors based on F^2^ are statistically about twice as large as those based on F, and R- factors based on ALL data will be even larger.

### Fractional atomic coordinates and isotropic or equivalent isotropic displacement parameters (Å^2^)

**Table:**

	*x*	*y*	*z*	*U*_iso_*/*U*_eq_	
Cd1	0.72922 (3)	0.36225 (2)	0.60671 (4)	0.02384 (16)	
Cd2	0.5000	0.5000	0.5000	0.02726 (18)	
Cl1	0.65520 (10)	0.45547 (8)	0.31268 (16)	0.0291 (3)	
Cl2	0.78296 (10)	0.26723 (9)	0.89644 (16)	0.0307 (3)	
Cl3	0.63396 (12)	0.47886 (9)	0.79095 (17)	0.0379 (3)	
Cl4	0.91217 (11)	0.42626 (9)	0.6250 (2)	0.0369 (3)	
N1	0.5591 (3)	0.2978 (3)	0.5599 (6)	0.0271 (9)	
N2	0.4709 (4)	0.3474 (3)	0.5015 (6)	0.0300 (9)	
N3	0.4303 (4)	0.2130 (3)	0.4573 (6)	0.0284 (9)	
N4	0.3704 (5)	0.1390 (3)	0.3952 (8)	0.0427 (12)	
N5	1.0964 (5)	0.2603 (4)	0.1439 (8)	0.0406 (11)	
N6	1.1503 (5)	0.3284 (4)	0.0808 (9)	0.0552 (15)	
N7	0.9940 (4)	0.3688 (3)	0.1463 (6)	0.0341 (10)	
N8	0.9074 (4)	0.4233 (3)	0.1599 (6)	0.0388 (11)	
C1	0.5314 (4)	0.2172 (3)	0.5326 (7)	0.0301 (10)	
H1	0.5755	0.1699	0.5613	0.036*	
C2	0.3953 (4)	0.2949 (4)	0.4392 (8)	0.0326 (11)	
H2	0.3273	0.3114	0.3895	0.039*	
C3	1.0035 (5)	0.2844 (4)	0.1816 (8)	0.0371 (12)	
H3	0.9528	0.2493	0.2253	0.044*	
C4	1.0865 (6)	0.3931 (4)	0.0848 (11)	0.0481 (16)	
O1W	0.1792 (5)	0.3900 (4)	0.5653 (10)	0.0663 (15)	
H4	1.103 (5)	0.450 (4)	0.066 (9)	0.038 (17)*	
H5	1.127 (6)	0.219 (5)	0.158 (11)	0.05 (2)*	
H8A	0.8524	0.4002	0.1015	0.050*	
H8B	0.9153	0.4271	0.2827	0.050*	
H4A	0.4298	0.0977	0.3612	0.050*	
H4B	0.350 (6)	0.120 (4)	0.516 (11)	0.045 (19)*	
HW2	0.237 (4)	0.396 (5)	0.638 (9)	0.07 (3)*	
HW1	0.142 (6)	0.435 (4)	0.580 (14)	0.11 (4)*	

### Atomic displacement parameters (Å^2^)

**Table:**

	*U*^11^	*U*^22^	*U*^33^	*U*^12^	*U*^13^	*U*^23^
Cd1	0.0255 (2)	0.0249 (2)	0.0204 (2)	0.00043 (12)	0.00023 (14)	0.00166 (12)
Cd2	0.0335 (3)	0.0216 (3)	0.0261 (3)	0.00527 (19)	0.0016 (2)	0.00027 (18)
Cl1	0.0363 (6)	0.0285 (6)	0.0229 (5)	0.0036 (5)	0.0046 (5)	0.0044 (4)
Cl2	0.0347 (6)	0.0341 (7)	0.0227 (6)	−0.0022 (5)	0.0013 (5)	0.0100 (5)
Cl3	0.0507 (8)	0.0372 (7)	0.0235 (6)	0.0143 (6)	−0.0041 (5)	−0.0085 (5)
Cl4	0.0327 (7)	0.0366 (7)	0.0410 (7)	−0.0101 (5)	0.0027 (5)	−0.0007 (6)
N1	0.026 (2)	0.026 (2)	0.029 (2)	0.0019 (16)	0.0020 (16)	0.0019 (16)
N2	0.032 (2)	0.029 (2)	0.028 (2)	0.0029 (18)	0.0022 (17)	−0.0003 (17)
N3	0.040 (2)	0.025 (2)	0.022 (2)	−0.0059 (18)	0.0095 (17)	−0.0032 (15)
N4	0.054 (3)	0.034 (3)	0.042 (3)	−0.020 (2)	0.011 (2)	−0.009 (2)
N5	0.046 (3)	0.034 (3)	0.041 (3)	−0.001 (2)	0.003 (2)	0.000 (2)
N6	0.054 (3)	0.047 (3)	0.071 (4)	0.002 (3)	0.031 (3)	0.005 (3)
N7	0.035 (2)	0.043 (3)	0.025 (2)	−0.0025 (19)	0.0072 (18)	−0.0042 (18)
N8	0.044 (3)	0.034 (2)	0.040 (3)	−0.002 (2)	0.009 (2)	−0.006 (2)
C1	0.034 (3)	0.025 (2)	0.031 (3)	0.003 (2)	0.002 (2)	0.002 (2)
C2	0.030 (3)	0.034 (3)	0.034 (3)	−0.001 (2)	0.003 (2)	−0.001 (2)
C3	0.042 (3)	0.037 (3)	0.031 (3)	−0.007 (2)	0.001 (2)	0.007 (2)
C4	0.051 (4)	0.037 (3)	0.061 (4)	−0.007 (3)	0.023 (3)	0.000 (3)
O1W	0.057 (3)	0.043 (3)	0.095 (5)	−0.011 (3)	−0.002 (3)	−0.002 (3)

### Geometric parameters (Å, º)

**Table:**

Cd1—N1	2.365 (4)	N4—H4A	1.0400
Cd1—Cl4	2.5120 (14)	N4—H4B	1.01 (8)
Cd1—Cl2	2.6148 (13)	N5—C3	1.299 (9)
Cd1—Cl3	2.6418 (14)	N5—N6	1.369 (8)
Cd1—Cl2^i^	2.6754 (13)	N5—H5	0.75 (8)
Cd1—Cl1	2.6769 (14)	N6—C4	1.292 (9)
Cd2—N2^ii^	2.393 (5)	N7—C3	1.336 (7)
Cd2—N2	2.393 (5)	N7—C4	1.362 (8)
Cd2—Cl3	2.5874 (16)	N7—N8	1.399 (7)
Cd2—Cl3^ii^	2.5875 (16)	N8—H8A	0.850
Cd2—Cl1	2.6332 (14)	N8—H8B	0.900
Cd2—Cl1^ii^	2.6333 (14)	C1—H1	0.9300
N1—C1	1.306 (7)	C2—H2	0.9300
N1—N2	1.382 (6)	C3—H3	0.9300
N2—C2	1.297 (7)	C4—H4	0.92 (6)
N3—C1	1.335 (7)	O1W—HW2	0.862 (10)
N3—C2	1.346 (7)	O1W—HW1	0.857 (10)
N3—N4	1.419 (6)		
			
N1—Cd1—Cl4	174.37 (11)	N1—N2—Cd2	115.6 (3)
Cl4—Cd1—Cl3	100.40 (5)	C1—N3—C2	106.4 (4)
Cl2—Cd1—Cl3	93.13 (5)	C1—N3—N4	128.4 (5)
N1—Cd1—Cl2^i^	83.80 (11)	C2—N3—N4	125.0 (5)
Cl4—Cd1—Cl2^i^	91.57 (5)	N3—N4—H4A	102.00
Cl2—Cd1—Cl2^i^	89.53 (3)	N3—N4—H4B	98 (4)
N1—Cd1—Cl1	83.54 (11)	H4A—N4—H4B	108.00
Cl4—Cd1—Cl1	93.40 (5)	C3—N5—N6	110.8 (5)
Cl2—Cd1—Cl1	174.60 (4)	C3—N5—H5	133 (6)
Cl3—Cd1—Cl1	84.86 (4)	N6—N5—H5	116 (6)
Cl2^i^—Cd1—Cl1	91.39 (4)	C4—N6—N5	104.5 (6)
N2^ii^—Cd2—Cl3	92.46 (11)	C3—N7—C4	106.0 (5)
N2—Cd2—Cl3	87.54 (12)	C3—N7—N8	129.0 (5)
N2^ii^—Cd2—Cl3^ii^	87.54 (11)	C4—N7—N8	125.0 (5)
N2—Cd2—Cl3^ii^	92.46 (12)	N7—N8—H8A	108.00
N2^ii^—Cd2—Cl1	97.50 (11)	N7—N8—H8B	97.00
N2—Cd2—Cl1	82.50 (11)	H8A—N8—H8B	121.00
Cl3—Cd2—Cl1	86.85 (5)	N1—C1—N3	109.7 (5)
Cl3^ii^—Cd2—Cl1	93.15 (5)	N1—C1—H1	125.2
N2^ii^—Cd2—Cl1^ii^	82.50 (11)	N3—C1—H1	125.2
N2—Cd2—Cl1^ii^	97.50 (11)	N2—C2—N3	109.7 (5)
Cl3—Cd2—Cl1^ii^	93.15 (5)	N2—C2—H2	125.1
Cl3^ii^—Cd2—Cl1^ii^	86.85 (5)	N3—C2—H2	125.1
Cd2—Cl1—Cd1	85.79 (4)	N5—C3—N7	107.6 (5)
Cd1—Cl2—Cd1^iii^	146.79 (5)	N5—C3—H3	126.2
Cd2—Cl3—Cd1	87.44 (4)	N7—C3—H3	126.2
C1—N1—N2	107.0 (4)	N6—C4—N7	111.1 (6)
C1—N1—Cd1	130.5 (3)	N6—C4—H4	126 (4)
N2—N1—Cd1	120.0 (3)	N7—C4—H4	122 (4)
C2—N2—N1	107.2 (4)	HW2—O1W—HW1	106 (3)
C2—N2—Cd2	136.6 (4)		

Symmetry codes: (i) *x*, −*y*+1/2, *z*−1/2; (ii) −*x*+1, −*y*+1, −*z*+1; (iii) *x*, −*y*+1/2, *z*+1/2.

### Hydrogen-bond geometry (Å, º)

**Table:**

*D*—H···*A*	*D*—H	H···*A*	*D*···*A*	*D*—H···*A*
O1*W*^ii^—H*W*2^ii^···Cl1	0.86 (6)	2.68 (7)	3.239 (6)	124 (6)
N4^iv^—H4*B*^iv^···Cl3	1.00 (8)	2.60 (7)	3.399 (5)	136 (5)
N8^v^—H8*A*^v^···Cl2	0.85	2.64	3.370 (5)	144
O1*W*^ii^—H*W*1^ii^···Cl4	0.86 (7)	2.67 (8)	3.319 (6)	134 (8)
N8—H8*B*···Cl4	0.90	2.53	3.423 (5)	172
N5^vi^—H5^vi^···O1*W*	0.75 (8)	1.97 (8)	2.649 (8)	151 (8)
O1*W*—H*W*2···N4^iii^	0.86 (6)	2.44 (6)	3.247 (9)	157 (6)

Symmetry codes: (ii) −*x*+1, −*y*+1, −*z*+1; (iii) *x*, −*y*+1/2, *z*+1/2; (iv) −*x*+1, *y*+1/2, −*z*+3/2; (v) *x*, *y*, *z*+1; (vi) *x*−1, −*y*+1/2, *z*+1/2.

## References

[bb1] Brandenburg, K. (2006). *DIAMOND*. Crystal Impact GbR, Bonn, Germany.

[bb2] Choi, K. Y. & Jeon, Y. M. (2003). *Inorg. Chem. Commun.* **6**, 1294–1296.

[bb3] Enraf–Nonius (1994). *CAD-4 EXPRESS*. Enraf–Nonius, Delft, The Netherlands.

[bb4] Gao, C., Wu, Y.-Z., Gong, H.-B., Hao, X.-P., Xu, X.-G. & Jiang, M.-H. (2008). *Inorg. Chem. Commun.* **11**, 985–987.

[bb5] Groom, C. R., Bruno, I. J., Lightfoot, M. P. & Ward, S. C. (2016). *Acta Cryst.* B**72**, 171–179. 10.1107/S2052520616003954 PMC4822653 27048719

[bb6] Guo, F., Zhu, B., Xu, G., Zhang, M., Zhang, X. & Zhang, J. (2013). *J. Solid State Chem.* **199**, 42–48.

[bb7] Haasnoot, J. (2000). *Coord. Chem. Rev.* **200–202**, 131–185.

[bb8] Harms, K. & Wocadlo, S. (1995). *XCAD4*. Program for Processing CAD-4 Diffractometer Data. University of Marburg, Germany.

[bb9] Li, C. P. & Du, M. (2011). *Inorg. Chem. Commun.* **14**, 502–513.

[bb10] Liao, J., Lai, C., Ho, C. & Su, C. (2004). *Inorg. Chem. Commun.* **7**, 402–404.

[bb11] North, A. C. T., Phillips, D. C. & Mathews, F. S. (1968). *Acta Cryst.* A**24**, 351–359.

[bb12] Sheldrick, G. M. (2008). *Acta Cryst.* A**64**, 112–122. 10.1107/S0108767307043930 18156677

[bb13] Sheldrick, G. M. (2015). *Acta Cryst.* C**71**, 3–8.

[bb14] Su, Y., Goforth, A., Smith, M. & zur Loye, H. (2003). *Inorg. Chem.* **42**, 5685–5692. 10.1021/ic034388l 12950218

[bb15] Tao, J., Tong, M. L. & Chen, X. M. (2000). *J. Chem. Soc. Dalton Trans.* pp. 3669–3674.

[bb16] Wang, P.-N., Yeh, C.-W., Tsou, C.-H., Ho, Y.-W., Lee, H.-T. & Suen, M.-C. (2014). *Inorg. Chem. Commun.* **43**, 70–74.

[bb17] Westrip, S. P. (2010). *J. Appl. Cryst.* **43**, 920–925.

[bb18] Xiong, R.-G., You, X.-Z., Abrahams, B. F., Xue, Z. & Che, C.-M. (2001). *Angew. Chem. Int. Ed.* **40**, 4422–4425. 10.1002/1521-3773(20011203)40:23<4422::aid-anie4422>3.0.co;2-g 12404434

[bb19] Xuan-Wen, L. (2005). *Acta Cryst.* E**61**, m1777–m1778.

[bb20] Zhai, Q.-G., Wu, X.-Y., Chen, S.-M., Lu, C.-Z. & Yang, W.-B. (2006). *Cryst. Growth Des.* **6**, 2126–2135.

